# Deep learning‐based body weight from scout images can be an alternative to actual body weight in CT radiation dose management

**DOI:** 10.1002/acm2.14080

**Published:** 2023-06-19

**Authors:** Shota Ichikawa, Hideki Itadani, Hiroyuki Sugimori

**Affiliations:** ^1^ Graduate School of Health Sciences Hokkaido University Sapporo Japan; ^2^ Department of Radiological Technology Kurashiki Central Hospital Kurashiki Okayama Japan; ^3^ Faculty of Health Sciences Hokkaido University Sapporo Japan

**Keywords:** body weight, CT dose index, deep learning, diagnostic reference levels, dose–length product, size‐specific dose estimates

## Abstract

**Purpose:**

Accurate body weight measurement is essential to promote computed tomography (CT) dose optimization; however, body weight cannot always be measured prior to CT examination, especially in the emergency setting. The aim of this study was to investigate whether deep learning‐based body weight from chest CT scout images can be an alternative to actual body weight in CT radiation dose management.

**Methods:**

Chest CT scout images and diagnostic images acquired for medical checkups were collected from 3601 patients. A deep learning model was developed to predict body weight from scout images. The correlation between actual and predicted body weight was analyzed. To validate the use of predicted body weight in radiation dose management, the volume CT dose index (CTDI_vol_) and the dose–length product (DLP) were compared between the body weight subgroups based on actual and predicted body weight. Surrogate size‐specific dose estimates (SSDEs) acquired from actual and predicted body weight were compared to the reference standard.

**Results:**

The median actual and predicted body weight were 64.1 (interquartile range: 56.5–72.4) and 64.0 (56.3–72.2) kg, respectively. There was a strong correlation between actual and predicted body weight (*ρ* = 0.892, *p* < 0.001). The CTDI_vol_ and DLP of the body weight subgroups were similar based on actual and predicted body weight (*p* < 0.001). Both surrogate SSDEs based on actual and predicted body weight were not significantly different from the reference standard (*p* = 0.447 and 0.410, respectively).

**Conclusion:**

Predicted body weight can be an alternative to actual body weight in managing dose metrics and simplifying SSDE calculation. Our proposed method can be useful for CT radiation dose management in adult patients with unknown body weight.

## 1 INTRODUCTION

Computed tomography (CT) scan plays an important role in the screening, diagnosis, and management of patients. However, increased radiation exposure is a concern due to the potential risk of radiation‐induced malignancies. In Japan, since April 2020, the safety management of medical exposure has been enforced due to the partial revision of the Enforcement Regulations of the Medical Care Law. This has promoted the recording and management of CT radiation doses and the increased use of dose monitoring systems in clinical practice.

The volume CT dose index (CTDI_vol_) and the dose–length product (DLP) outputted by the CT scanner are the two most common dose metrics routinely used in clinical practice. Diagnostic reference levels (DRLs), which are the benchmarks for optimizing patient imaging, are established based on the national collection of both parameters for specific types of CT scan examinations performed with standard sizes.^1^, ^2^, ^3^, ^4^ When establishing DRLs, the standardization of patient size has traditionally been accomplished by weight restriction.^5^ Based on the Japanese DRLs, the standard size is defined as a body weight of 50−70 kg.^1^ Since DRLs for adult patients vary widely with patient size, setting DRLs only for patients of standard sizes is not appropriate.^6^ Accurate measurement of body weight is essential to promote CT dose optimization by comparing dose values in one's own institution with those of the DRLs, and to establish size‐based reference doses.

More recently, the American Association of Physicists in Medicine (AAPM) has proposed the use of size‐specific dose estimates (SSDEs) to more accurately assess an individual patient's dose by taking into account the patient's body size.^7^, ^8^ This is because CTDI_vol_ is delivered by a specific standard phantom size and cannot provide the actual radiation dose absorbed by the individual patient.^9^ SSDE is calculated from CTDI_vol_ by applying a conversion factor related to patient size expressed as an effective diameter (D_eff_) or water‐equivalent diameter (D_w_). D_eff_ is calculated using the measured anteroposterior and/or lateral patient dimensions, whereas D_w_ takes into account tissue attenuation in addition to the geometric size of the patient.^8^ AAPM Task Group 220 has recommended that both patient size and SSDE should be calculated in a robust and consistent manner across CT scanner manufacturers. The resulting values should be stored either in the Digital Imaging and Communications in Medicine (DICOM) image header or in the DICOM‐structured dose report.^8^ However, at a practical level, these metrics are not yet available in a structured radiation dose report. Therefore, the use of these metrics can be cumbersome and time‐consuming, and it requires commercially available radiation dose monitoring software. In addition, an accurate calculation is challenging to achieve in cases in which the whole‐body circumference is outside the field of view, which is common in CT scans of obese patients and in cardiac and musculoskeletal CT scans. Therefore, several studies have focused on simplifying the calculation of SSDE using the correlations between D_eff_/D_w_ and body weight or body mass index.^10^, ^11^, ^12^, ^13^, ^14^

Based on the above situation, body weight is an indispensable size metric to promote CT dose optimization in clinical practice. However, body weight cannot always be measured prior to CT examination, especially in the emergency setting. As a result, body weight is not always recorded in the DICOM tags, making it impossible to use the scanned data for comparing dose values in one's own institution with those of the DRLs. In addition, for patients with unknown body weight, it becomes difficult to determine whether an appropriate individual patient's dose based on their size was delivered.

Body size estimation has attracted a lot of attention in several papers. Geraghty et al. used the anatomical structures outlined in the CT section including L1 to generate equations for predicting body weight. However, this is extremely complex and time‐consuming.^15^ Gascho et al. developed a linear regression equation based on effective mAs from CT dose modulation on whole‐body scans. However, it was limited to polytrauma patients undergoing whole‐body CT scans.^16^ A few studies have presented surrogate metrics of body weight for CT dose management. Fukunaga et al. demonstrated the usefulness of D_eff_ as a somatometric parameter for CT dose management in adult patients of unknown body weight.^17^ Li et al. expressed body habitus in terms of T‐shirt size and provided dose reference values in patients with different T‐shirt sizes.^18^ However, compared to body weight, these metrics lose an intuitive sense of size for radiologists and radiological technologists. A previous study showed that body weight can be predicted from CT scout images using deep learning.^19^ We hypothesized that predicted body weight from CT scout images may be useful for CT dose management in patients with unknown body weight. The current study aimed to investigate whether deep learning‐based body weight from chest CT scout images can be an alternative to actual body weight in CT dose management.

## 2 METHODS

Part of our patient population overlaps with a previous report.^19^ A previous study aimed to develop a convolutional neural network‐based method using chest and abdominal CT scout images for predicting body weight and to evaluate the correlation between actual and predicted body weight. Meanwhile, the current study investigated whether deep learning‐based body weight from chest CT scout images can be an alternative to actual body weight in CT radiation dose management by evaluating the relationships between body weight and dose metrics.

### 2.1 Population and CT acquisition

This retrospective study was approved by the institutional review board, and the need for individual informed consent was waived due to the retrospective nature of the study.

Inclusion criteria were as follows: (1) patients with chest CT acquisition for medical checkups between June 2019 and May 2022, (2) those with body weight information obtained using a calibrated scale on the same day of the CT acquisition, and (3) those with available information on dose metrics derived using the radiation dose monitoring software (Radimetrics; Bayer HealthCare, Whippany, New Jersey, USA). A total of 3601 CT scans were obtained, and the constructed datasets were randomly divided into the training (*n* = 2520), validation (*n* = 360), and test (*n* = 721) subsets.

CT scan images were obtained using an 80‐detector row CT scanner (Aquilion Prime SP; Canon Medical Systems, Otawara, Japan). The frontal view of the chest CT scout images was acquired using the following parameters: tube voltage, 120 kVp; tube current, 10 mA; and field of view, 500 mm. The chest CT scan was performed with the following parameters: tube voltage, 120 kVp; tube current, automatic tube current modulation technique (slice thickness, 5 mm; reconstruction kernel, FC03; and tube current range, 10−150 mA); reconstruction kernels, FC03 and FC52; reconstruction method, adaptive iterative dose reduction using three‐dimensional standard; beam collimation, 1.0 mm × 40 rows; pitch factor, 1.475; slice thickness, 5 mm; and field of view, 320 mm, but it varied in some cases depending on the patient's physique. Our body weight prediction method is based solely on two‐dimensional radiographic scout images taken prior to the CT scan, while dose metrics were calculated using the diagnostic CT scan data.

### 2.2 Deep learning method for body weight prediction

**FIGURE 1:**
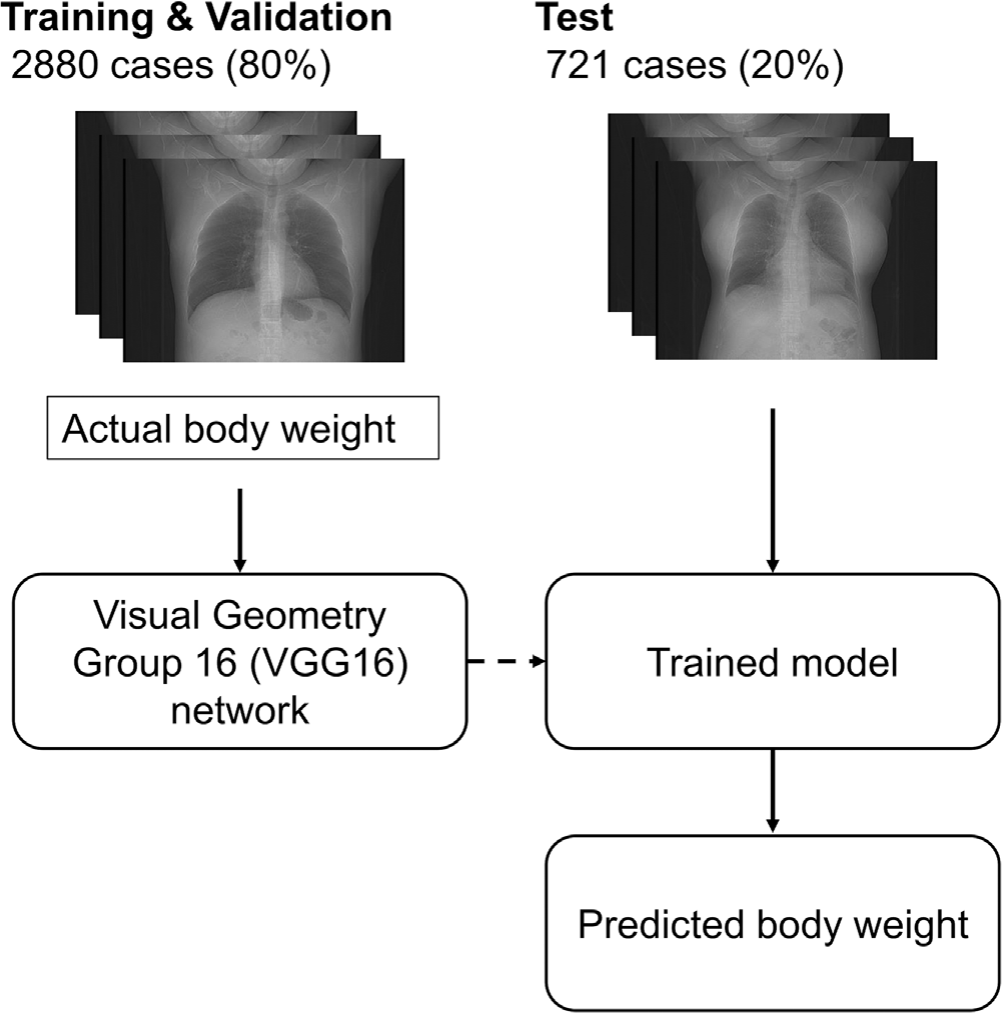
Overview of the deep learning model for body weight prediction.

In a previous report, the principle of predicting body weight from CT scout images using the deep learning technique was demonstrated.^19^ Briefly, supervised training of a convolutional neural network was performed using chest CT scout images as input data and the corresponding body weight as reference data (Figure 1). The body weight prediction model was based on the Visual Geometry Group 16 (VGG16) architecture.^20^ The final layers of the original VGG16 models were replaced as follows: (1) a linear layer with an output size of 512, (2) a rectified linear unit function, (3) a dropout (rate: 0.4), and (4) a linear layer with an output size of 1. The loss function was the mean squared error, and the Adam optimizer was used to adjust the model weights. The initial learning rate was 0.001. The maximum number of training epochs was 50, and the batch size was 128. The framework used was PyTorch Lightning 1.6.4. The GPU was an NVIDIA GeForce RTX 3090 Ti 24 GB. The model was trained using the training and validation datasets, and the remaining test dataset was used to evaluate the prediction accuracy and subsequently validate the use of predicted body weight in radiation dose management.

### 2.3 Relationships between body weight and CTDIvol/DLP

CTDI_vol_ and DLP in diagnostic scans were collected from a structured radiation dose report. To validate the use of predicted body weight in radiation dose management, we evaluated the relationships between actual body weight and CTDI_vol_/DLP and between predicted body weight and CTDI_vol_/DLP. In addition, we grouped the patients into subgroups based on 10 kg intervals, taking into account the previous version of the Japanese DRLs,^21^ which defined the standard size as a body weight of 50−60 kg for CT examinations, except for coronary CT angiography. We considered the previous version of the DRLs because the range of standard size was narrower (50−60 kg) compared to the current version (50−70 kg). Briefly, patients were divided into subgroups (≤ 50 , 50−60 , 60−70 , 70−80 , and ≥ 80 kg) based on their actual or predicted body weight. Due to the limited size of our dataset, we were unable to further subgroup the patients. CTDI_vol_ and DLP were compared between subgroups based on actual and predicted body weight.

### 2.4 Relationships between body weight and SSDE

SSDE was calculated by multiplying CTDI_vol_ by a size‐dependent conversion factor ($\;f_{D_{eff}}$) based on D_eff_ as follows:

(1) $$\begin{array}{c} SSDE\;=CTDI_{vol}\times \;f_{D_{eff}}\; \end{array}$$

According to the AAPM Report 204,^7^ the f_Deff_ for converting CTDI_vol_ to SSDE was obtained using the following equation:

(2) $$\begin{array}{c} \;f_{D_{eff}}=\;3.704369\times \;e^{-0.03672\times D_{eff}} \end{array}$$

As a reference standard, D_eff_ was automatically calculated from the lateral diameter on CT scout images by Radimetrics (D_eff_reference_). In addition, regression equations for D_eff_reference_ and actual body weight, and D_eff_reference_ and predicted body weight were used to determine surrogate D_eff_ values (D_eff_actual_ and D_eff_predicted_)_,_ as described in a previous report.^11^ The values for either actual or predicted body weight were inserted into the regression equations to calculate the corresponding D_eff_actual_ and D_eff_predicted_. SSDEs were calculated using the corresponding conversion factor for D_eff_reference_, D_eff_actual,_ and D_eff_predicted_. The surrogate SSDEs (SSDE_actual_ and SSDE_predicted_) were compared to the reference standard (SSDE_reference_).

### 2.5 Statistical analyses

R version 3.5.1 (R Foundation for Statistical Computing, Vienna, Austria) was used for all statistical analyses. A *p*‐value of < 0.05 was considered statistically significant. Shapiro–Wilk tests were performed to assess the normality of the data.

The mean absolute error (MAE) and Spearman's rank correlation coefficient (ρ) were calculated to assess the accuracy of body weight prediction. Linear regression models were used to estimate the relationships between body weight and dose metrics (CTDI_vol_ and DLP) and between body weight and body diameter (D_eff_). Correlations between the two metrics were examined using Spearman's rank correlation coefficient (ρ). The Wilcoxon rank‐sum test was used to compare dose metrics (CTDI_vol_, DLP, and SSDE) between groups. The equivalence test (two one‐sided tests) was also used to assess whether the dose metrics of the groups based on predicted body weight were equivalent to those of the groups based on actual body weight. For the equivalence test, the margin of equivalence was set within the standard deviation of the data.

## 3 RESULTS

Note that the results presented below are based on the test sets only.

### 3.1 Accuracy of predicted body weight

**FIGURE 2:**
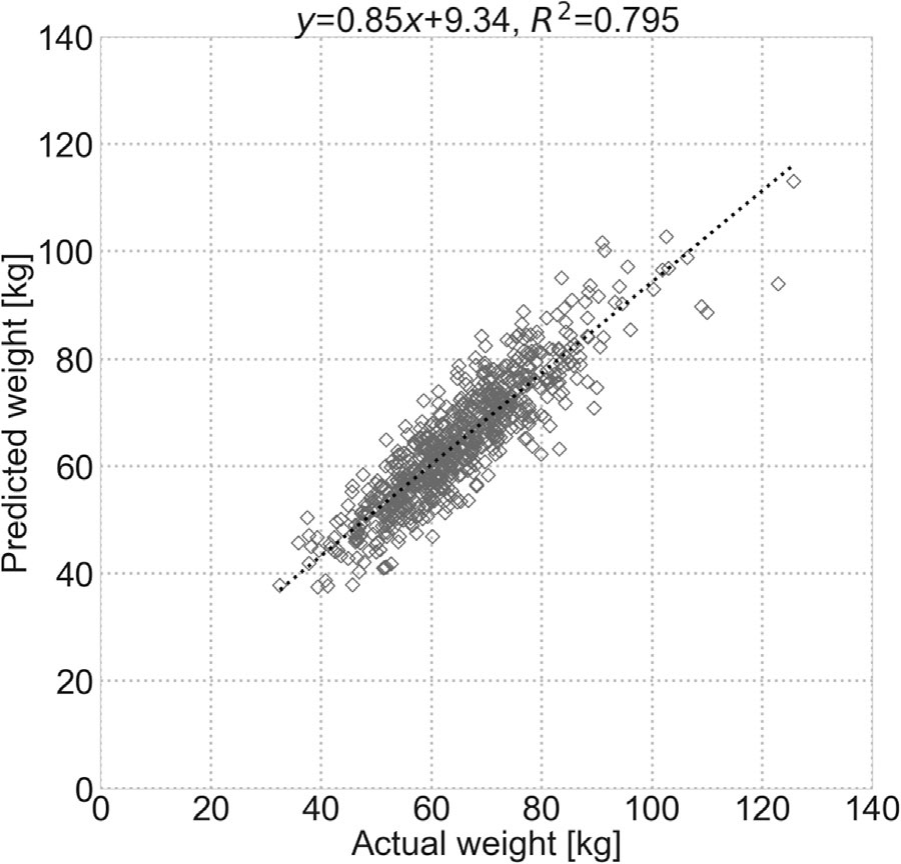
Scatter plot comparing actual and predicted body weight.

Table 1 shows the demographic characteristics of the patients. The median actual body weight of the patients in the test sets was 64.1 (interquartile range [IQR]: 56.5−72.4) kg. Their predicted body weight was 64.0 (56.3−72.2) kg. The MAE of the body weight prediction model was 4.41 kg. Figure 2 shows the scatterplot comparing actual and predicted body weight. There was a strong correlation between actual and predicted body weight (*ρ* = 0.892, *p* < 0.001).

**TABLE 1:** Demographics of patients included in this study.

Characteristics	Training	Validation	Test
*N*	2520	360	721
Sex, male/female	1800/720	262/98	513/208
Age, median (IQR) years	60.9 (51.9−69.7)	60.9 (52.1−70.2)	60.5 (49.3−69.2)
Height, median (IQR) (cm)	166.7 (160.0−171.9)	166.8 (160.2−172.4)	167.1 (160.3−171.7)
Weight, median (IQR) (kg)	64.1 (56.9−72.1)	63.9 (57.2−72.3)	64.1 (56.5−72.4)
BMI, median (IQR) (kg/m^2^)	23.2 (21.3−25.4)	23.2 (21.3−25.5)	23.2 (21.2−25.5)

Abbreviations: BMI, body mass index; IQR, interquartile range.

### 3.2 Relationships between body weight and CTDIvol/DLP

**FIGURE 3:**
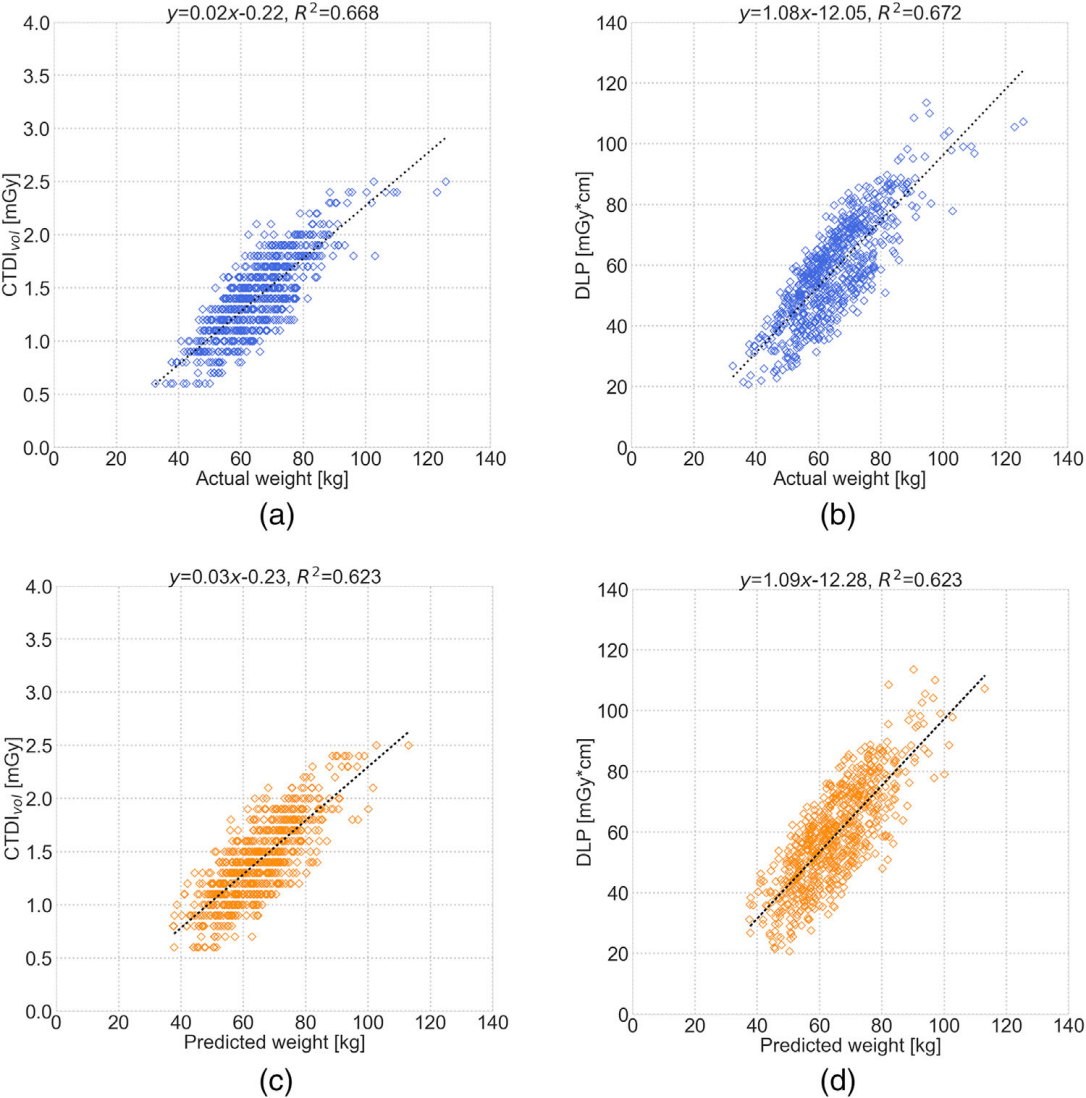
Scatter plots showing the relationships between body weight and CT dose metrics: (a) actual body weight versus volume CT dose index (CTDI_vol_), (b) actual body weight versus dose–length product (DLP), (c) predicted body weight versus CTDI_vol_, and (d) predicted body weight versus DLP.

Figure 3 shows the scatterplots showing the relationships between actual body weight and CTDI_vol_/DLP, and between predicted body weight and CTDI_vol_/DLP. The correlation coefficient for CTDI_vol_ was 0.792 (*p* < 0.001) and 0.771 (*p* < 0.001) for actual and predicted body weight, respectively. The correlation coefficient for DLP was 0.792 (*p* < 0.001) and 0.770 (*p* < 0.001) for actual and predicted body weight, respectively.

Table 2 shows the median (IQR) CTDI_vol_ and DLP in the body weight subgroups. There were no significant differences in CTDI_vol_ and DLP in all subgroups. CTDI_vol_ and DLP based on actual and predicted body weight were similar in all subgroups (*p* < 0.001) (Table 3).

**TABLE 2:** Comparison of CTDIvol and DLP across body weight subgroups.

	Actual weight		Predicted weight		*p*
CTDI_vol_/DLP	*N*	Median (IQR)	*N*	Median (IQR)	
CTDI_vol_ (mGy)					
Weight ≤ 50 kg	65	0.9 (0.8−1.0)	64	0.9 (0.8−1.1)	0.334
Weight 50−60 kg	192	1.2 (1.0−1.3)	202	1.2 (1.0−1.3)	0.652
Weight 60−70 kg	222	1.4 (1.2−1.6)	228	1.4 (1.2−1.6)	0.393
Weight 70−80 kg	168	1.7 (1.4−1.8)	158	1.7 (1.5−1.8)	0.299
Weight ≥ 80 kg	74	2.0 (1.8−2.2)	69	1.9 (1.8−2.2)	0.490
DLP (mGy·cm)					
Weight ≤ 50 kg	65	36.2 (31.7−42.2)	64	37.5 (32.0−43.3)	0.519
Weight 50−60 kg	192	49.3 (41.2−56.0)	202	48.8 (40.7−56.0)	0.865
Weight 60−70 kg	222	59.6 (49.9−66.1)	228	59.7 (50.7−67.7)	0.484
Weight 70−80 kg	168	70.8 (58.7−75.5)	158	71.0 (60.9−77.5)	0.255
Weight ≥ 80 kg	74	84.4 (76.3−88.6)	69	82.6 (71.5−88.6)	0.255

*Note*: The Wilcoxon rank‐sum test was used to calculate the *p*‐value.

Abbreviations: CTDI_vol_, volume computed tomography dose index; DLP, dose–length product; IQR, interquartile range.

**TABLE 3:** Results of the equivalence test between groups based on actual and predicted body weight for CTDIvol and DLP.

CTDI_vol_/DLP	Equivalence margin	95% CI	*p*
CTDI_vol_ (mGy)			
Weight ≤ 50 kg		−0.090 to 0.018	<0.001
Weight 50−60 kg		−0.030 to 0.046	<0.001
Weight 60−70 kg	0.375	−0.061 to 0.018	<0.001
Weight 70−80 kg		−0.073 to 0.014	<0.001
Weight ≥ 80 kg		−0.034 to 0.124	<0.001
DLP (mGy·cm)			
Weight ≤ 50 kg		−3.443 to 1.149	<0.001
Weight 50−60 kg		−1.471 to 1.757	<0.001
Weight 60−70 kg	16.248	−2.560 to 0.822	<0.001
Weight 70−80 kg		−3.379 to 0.378	<0.001
Weight ≥ 80 kg		−1.259 to 6.309	<0.001

*Note*: The equivalence test (two one‐sided tests) was used to calculate the *p*‐value.

Abbreviations: CI, confidence interval; CTDI_vol_, volume computed tomography dose index; DLP, dose–length product.

### 3.3 Relationships between body weight and SSDE

**FIGURE 4:**
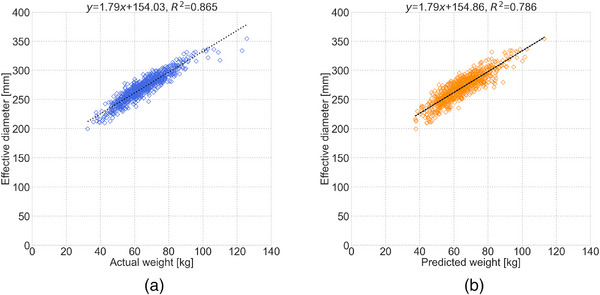
Scatter plots showing the relationships (a) between actual body weight and effective diameter (D_eff_) and (b) between predicted body weight and D_eff_.

Figure 4 shows the scatterplots showing the relationships between actual body weight and D_eff_reference_, and between predicted body weight and D_eff_reference_. There was a significant correlation between actual body weight and D_eff_reference_ (*ρ* = 0.927, *p* < 0.001). There was also a correlation between predicted body weight and D_eff_reference_ (*ρ* = 0.880, *p* < 0.001). The median (IQR) D_eff_reference_, D_eff_actual_, and D_eff_predicted_ were 270.8 (254.4−285.4), 268.8 (255.2−283.6), and 269.4 (255.6−284.1) mm, respectively.

The median SSDE_reference_, SSDE_actual_, and SSDE_predicted_ were 1.90 (1.62−2.19), 1.90 (1.61−2.20), and 1.91 (1.61−2.21), respectively. SSDE_actual_ (*p* = 0.447) and SSDE_predicted_ (*p* = 0.410) were not significantly different from SSDE_reference_ (Table 4). The equivalence test showed that the surrogate SSDEs were equivalent to the reference standard (*p* < 0.001) (Table 5).

**TABLE 4:** Comparison of surrogate SSDEs based on body weight and the reference standard.

SSDE	Median (IQR)	*p*
SSDE_reference_	1.90 (1.62−2.19)	–
SSDE_actual_	1.90 (1.61−2.20)	0.447
SSDE_predicted_	1.91 (1.61−2.21)	0.410

*Note*: The Wilcoxon rank‐sum test was used to calculate the *p*‐value.

Abbreviations: IQR, interquartile range; SSDE, size‐specific dose estimates.

**TABLE 5:** Results of the equivalence test between surrogate SSDEs based on body weight and the reference standard.

SSDE	Equivalence margin	95% CI	*p*
SSDE_reference_—SSDE_actual_	0.376	−0.036 to 0.031	<0.001
SSDE_reference_—SSDE_predicted_		−0.038 to 0.030	<0.001

*Note*: The equivalence test (two one‐sided tests) was used to calculate the *p*‐value.

Abbreviations: CI, confidence interval; SSDE, size‐specific dose estimates.

## 4 DISCUSSION

The current study aimed to investigate the feasibility of using predicted body weight obtained from chest CT scout images in CT dose management. Predicted body weight was strongly correlated with actual body weight. Moreover, actual and predicted body weight were comparable in managing dose metrics and simplifying the SSDE calculation. These results suggest that deep learning‐based body weight can be an alternative to actual body weight in CT dose management.

In certain situations, such as in the emergency setting, obtaining a patient's body weight can be challenging due to the limited availability of floor scales and patient responsiveness. As a result, scanned data of patients with unknown body weight must be excluded when comparing dose values in one's own institution with those of the DRLs. Moreover, it becomes difficult to ascertain whether the appropriate dose for individual patients based on their body size was delivered. To overcome these issues, we propose recording the predicted body weight from our method in the DICOM tags, which would promote CT dose management. Specifically, our proposed method could eliminate the need to exclude radiation dose data of patients with unknown body weight, enabling accurate radiation dose management and promoting the establishment of size‐based reference doses. Our approach provides an intuitive feeling and does not require manual measurement. It allows radiologists and radiological technologists to obtain the patient's body weight prior to a diagnostic scan and validate that the proper scanner output is programmed based on the patient's size. Knowledge of the patient's body attenuation or size characteristics prior to the diagnostic CT scan is helpful in tube voltage selection^22^ and personalized chest CT protocol optimization.^23^ In addition, body weight estimation from CT scout images is not affected by truncation because CT scout images are commonly acquired with an extended field of view compared to diagnostic scan images.

In terms of prediction accuracy, the coefficient of determination (*R* ^2^) between actual and predicted body weight in this study (*R* ^2^ = 0.795) was low compared to a previous study by Gascho et al., wherein *R* ^2^ was 0.938 with a simple equation using the correlation between body weight and effective mAs from CT dose modulation.^16^ This may be due to the fact that our work used only chest CT scout images to obtain the patient's body weight prior to the diagnostic scan. Meanwhile, Gascho et al. used whole‐body diagnostic scan data. Nevertheless, CTDI_vol_ and DLP were equivalent between the body weight subgroups based on actual and predicted body weight. In addition, comparable correlations were found between actual body weight and CTDI_vol_/DLP and between predicted body weight and CTDI_vol_/DLP. Therefore, the prediction accuracy of body weight may be sufficient for CT dose management considering patient size in clinical practice.

In terms of the relationships between body weight and D_eff_, our work (*ρ* = 0.927 and 0.880, *p* < 0.001 for actual and predicted weight, respectively) was comparable to that of previous studies. Boos et al. showed that D_eff_ was significantly correlated with body weight (*r* = 0.84, *p* < 0.05) on chest and abdominal CT scans.^11^ Fukunaga et al. showed a high correlation between body weight and D_eff_ on chest (*r* = 0.920 and 0.929, *p* < 0.05) and abdominal (*r* = 0.805, *p* < 0.05) CT scans.^17^ Kritsaneepaiboon et al. found a strong correlation (*r* = 0.943, *p* < 0.001) between body weight and D_eff_ on thoracic and abdominal CT scans.^13^ Based on our results and previous findings, body weight could be used as a surrogate parameter in the calculation of SSDE.

The current study had several limitations. First, the validation of our proposed method was performed only in the chest region. However, a previous study showed that body weight prediction from CT scout images using the deep learning model was successful not only in the chest but also in the abdomen.^19^ Thus, CT dose management using predicted body weight could be applied to the whole torso. Second, we explored the usefulness of our concepts using only the CT images of patients at a single institution. Thus, the ability of the method to generalize to CT images obtained at other institutions using machines from different manufacturers is unknown. However, it can be investigated with the method used in the current study. Finally, the domain of our proposed method was limited to an adult population because there were no pediatric patients in our datasets. In general, radiation exposure is of greater concern in pediatric patients than in adults. In fact, some studies have already established size‐specific DRLs for pediatric patients.^1^, ^24^, ^25^ Therefore, in the future, data from pediatric patients should be included in the training dataset, and the usefulness of predicted body weight for CT dose management in pediatric patients needs to be investigated.

## 5 CONCLUSION

Patient's body weight was predicted from chest CT scout images using a deep learning model. The feasibility of using predicted body weight in CT dose management was also investigated. The results showed that predicted body weight could be an alternative to actual body weight in managing dose metrics and simplifying SSDE calculation. Our proposed method can be useful for CT radiation dose management in adult patients with unknown body weight.

## AUTHOR CONTRIBUTIONS

Shota Ichikawa contributed to the study design, data collection, algorithm construction, and the writing and editing of the article. Hideki Itadani carried out the data collection and reviewing and editing of the article. Hiroyuki Sugimori performed supervision and reviewing and editing of the article. All authors read and approved the final manuscript.

## CONFLICT OF INTEREST STATEMENT

The authors report no conflicts of interest.

## Data Availability

The code generated during the current study is available from the corresponding author upon reasonable request. However, the authors do not currently have permission to provide image datasets. In order to provide data to a third‐party organization in any way, it is necessary to obtain approval from the Ethics Committee of the author institution after obtaining informed consent from patients and preparing an experimental plan that specifies the provision of data to the outside. Unfortunately, the authors cannot make the data publicly available in the prescribed form.
